# Transcriptomic analysis of gills provides insights into the molecular basis of molting in Chinese mitten crab (*Eriocheir sinensis*)

**DOI:** 10.7717/peerj.7182

**Published:** 2019-06-28

**Authors:** Jingjing Li, Jinsheng Sun, Xuewang Dong, Xuyun Geng, Gaofeng Qiu

**Affiliations:** 1 National Demonstration Center for Experimental Fisheries Science Education, Key Laboratory of Exploration and Utilization of Aquatic Genetic Resources, Ministry of Education, Key Laboratory of Freshwater Aquatic Genetic Resources, Ministry of Agriculture, Shanghai Ocean University, Shanghai, China; 2 Tianjin Diseases Prevention and Control Center of Aquatic Animals, Tianjin, China; 3 Tianjin Key Laboratory for Animal and Plant Resistance, College of Life Sciences, Tianjin Normal University, Tianjin, China

**Keywords:** Chitinase, Transcriptome, Chinese mitten crab (*Eriocheir sinensis*), Gill, Molting

## Abstract

Chinese mitten crab (*Eriocheir sinensis*) is an economically important freshwater aquaculture species and is a model species for research on the mechanism of molting. This study aimed to identify important candidate genes associated with the molting process and to determine the role of gills in the regulation of molting with the help of transcriptomic analysis. The transcriptomes of crabs at different molting stages—postmolt (PoM), intermolt (InM), premolt (PrM) and ecdysis (E)—were de novo assembled to generate 246,232 unigenes with a mean length of 851 bp. A total of 86,634 unigenes (35.18% of the total unigenes) were annotated against reference databases. Significantly upregulated genes were identified in postmolt compared to intermolt (1,475), intermolt compared to premolt (65), premolt compared to ecdysis (1,352), and ecdysis compared to postmolt (153), and the corresponding numbers of downregulated genes were 1,276, 32, 1,573 and 171, respectively. Chitin synthase, endochitinase, chitinase A, chitinase 3, chitinase 6 and chitin deacetylase 1 were upregulated during the postmolt and ecdysis stages, while phosphoglucomutase 3 (PGM3), glucosamine 6-phosphate deaminase (GNPDA) and glucosamine glycoside hydrolase (nagZ) were upregulated during the intermolt and premolt stages compared to the other stages. The upregulated genes were enriched in several lipid-related metabolic pathways, such as “fatty acid elongation”, “glycerophospholipid metabolism” and “sulfur metabolism”. Meanwhile, three signaling pathways, including the “phosphatidylinositol signaling system”, the “calcium signaling pathway” and the “GnRH signaling pathway” were also enriched. Tetraspanin-18, an important effector gene in the lysosomal pathway involved in cell apoptosis, up-regulate with the beginning of molting (in premolt stage) and reach the top in the ecdysis stage, and barely expressed in the intermolt stage. The expression variations in the tetraspanin-18 gene indicated that it may play an important role in the beginning of molting cycle, which might be regulated by the stress of salinity. This study revealed that the gills could participate in chitin degradation, in reestablishment of the exoskeleton and the signaling process. Based on transcriptomic analysis of the gills, we not only explored novel molecular mechanisms of molting in *E. sinensis* but also acquired foundational genetic data for *E. sinensis*.

## Introduction

*Eriocheir sinensis* or Chinese mitten crab, a kind of crab named for its furry claw feature, belongs to the taxa Arthropoda, Crustacea, Decapoda, Grapsidae and Eriocheir (Veilleux & De Lafontaine, 2007). In China, Chinese mitten crab (*E. sinensis*) is an economically important freshwater aquaculture species (Sui et al., 2009). *E. sinensis* originated from east Asia (Herborg et al., 2003) but is found in Europe and North America, where it is considered an invasive species, damaging river walls and embankments and competing with native species (Dittel & Epifanio, 2009; Ruiz et al., 2006). This species can live in freshwater and seawater, and the unique physiological characteristics of *E. sinensis* allow this species to inhabit rice fields by the sea and inland rivers; thus, this species is a perfect model for research on osmoregulation.

The life cycle of *E. sinensis* can be divided into five stages: The larvae hatch from the eggs in seawater and then form megalopae that are 3–4 mm in length, eventually growing into small mitten crabs. The crab shell is a vital tissue or organ that is an indurate structure and does not grow with body size. The crabs abandon the old crab shell and grow a new shell, and this entire essential biological process is known as molting. During the entire life cycle, molting occurs several times (up to 18 times or more) and is essential to the growth, development, and reproduction of *E. sinensis* (Abuhagr et al., 2014; Chung, Dircksen & Webster, 1999). The molting cycle of *E. sinensis* can be divided into four major stages based on observed morphological features (Shen et al., 2011): intermolt (InM), premolt (PrM), ecdysis (E) and postmolt (PoM). The InM is the longest stage that involves important physiological processes, such as energy accumulation and muscle regeneration. During the PrM stage, *E. sinensis* reabsorbs its old exoskeleton and forms a new one. After that, the crab absorbs a large amount of water, abandons the old skeleton, and forms and hardens a new soft exoskeleton as soon as possible via sclerotization and mineralization for defense and locomotion during the E and PoM stages. During molting, the crab is frail and unprotected; therefore, the molting process is strictly regulated.

The regulation of molting is under the strict control of the molting glands and hormones (Chang & Mykles, 2011). The molting glands (Y-organs or YOs), located at the front end of cephalothorax, secrete ecdysteroid hormone (EH). The sinus gland/X-organ complex (XO-SG), located in the eyestalk, stores the neuroendocrine hormones and the molt-inhibiting hormone (MIH), which can inhibit the molting process of crustaceans (Chen et al., 2012; Watson et al., 2001). The EH and MIH exhibit mutual antagonism in the regulation of the molting process. During molting, MIH binds to a membrane-bound MIH receptor and triggers signaling pathways involving cGMP, cAMP, or both (Nakatsuji, Lee & Watson, 2009). There are some other hormones associated with molting, including vitellogenesis-inhibiting hormone (VIH) (Zmora et al., 2009) and type I crustacean hyperglycemic hormone (CHH) (Jeon et al., 2012). In addition to the effects and regulatory roles of molting glands (XO-SG and YOs) and associated hormones, the roles of other tissues (e.g., chitin shell, hepatopancreas, eyestalk, gill) in this process should be considered. To investigate the roles of other tissues, the transcriptomes of other tissues were sequenced and analyzed. The transcriptome is the set of all RNA molecules in a cell or a population of cells.

Transcriptomic techniques include DNA microarrays and a high-throughput sequencing technology known as RNA-Seq. High-throughput sequencing has been used to identify molting-related genes, to identify previously unknown molecular mechanisms of molting regulation, and determine the roles of novel genes associated with different molting stages (De Kleijn & Van Herp, 1995). Analysis of different tissues has revealed some important novel genes and pathways. Transcriptomic analyses of the chitin shells of *Euphausia superba* and *Portunus pelagicus* at different stages of the molting cycle were carried out, and differentially expressed genes (DEGs), most of which associated with cuticle formation and phenotypic structural changes, were identified (Kuballa, Merritt & Elizur, 2007; Seear et al., 2010). Transcriptomic profilings of the hepatopancreas of *E. sinensis* and *Litopenaeus vannamei* have led to the identification of DEGs and provided insights into the hepatopancreas in energy metabolism and biological processes pertaining to molting (Huang et al., 2015; Zeng et al., 2013). Transcriptomic analyses of the eyestalk of *Portunus trituberculatus* during the molting cycle were performed, which observed regulations of neuromodulator-related pathways and other important genes (Lv et al., 2017; Xu et al., 2015). These studies provided foundations for research on the functions and regulations of different tissues in the molting process.

Although many of the studies mentioned above were performed to elucidate the molecular and physiological mechanisms underlying molting in crustaceans, the studies conducted to date have focused on a few main tissues. The gills of crustaceans are multifunctional organs (Henry et al., 2012). The multifunctional gills and the excretory organs play important roles in osmoregulation and ionic regulation in crustaceans (Freire, Onken & McNamara, 2008; Zhou & Jiang, 2004). The gill itself is a tissue that comes in direct contact with the water in the environment of the organism. *E. sinensis* can live in exterior and interior of the water body. The molting cycle is a complex process that is regulated by many environmental factors, such as temperature, salinity, light and pH. Gills can sense changes in these environmental factors and perform osmoregulatory functions (Zhou & Jiang, 2004). Due to the multifunctionality of this organ, gill tissue may be important for research on the regulation of molting cycles (Gross et al., 2001). To elucidate the important role of gills in the molting cycle of *E. sinensis*, the transcriptomes of gills at four different molting stages (InM, PrM, E and PoM) were sequences and analyzed by RNA-Seq. This study aimed to identify important candidate genes associated with or playing regulatory roles in the molting process and to reveal the novel role of gills in the regulation of molting, not only allowing us to explore additional molecular mechanisms underlying molting in *E. sinensis* but also providing foundational genetic data for *E. sinensis*.

## Materials & Methods

### Sample preparation

Healthy one-year-old *E. sinensis* crabs were randomly captured from Xieyuan Fishing Company in Qilihai region in Tianjin city, China. The crabs were acclimatized in plastic tanks with adequate aeration and at an appropriate temperature (25 °C) and provided food twice daily for one week before sampling. Three individual crabs were sampled in each molting stage: postmolt (PoM, stage A), intermolt (InM, stage C), premolt (PrM, stage D) and ecdysis (stage E), as identified by a molt stage examination method described in a previous study (Tian, Kang & Mu, 2012). Fresh gill tissues from each crab, anesthetized on ice, were rapidly collected and immediately stored in liquid nitrogen for RNA isolation.

### RNA isolation, library preparation and transcriptome sequencing

Total RNA was extracted from each sample by using TRIzol reagent (Invitrogen, CA, USA) according to the manufacturer’s instructions. RNA degradation and contamination were monitored on 1% agarose gels, and purity was checked using a NanoPhotometer® spectrophotometer (Implen, CA, USA). The concentration and integrity of the total RNA were assessed using the Qubit® RNA Assay Kit with a Qubit® 2.0 fluorometer (Life Technologies, CA, USA) and the RNA Nano 6000 Assay Kit for the Agilent Bioanalyzer 2100 system (Agilent Technologies, CA, USA).

Sequencing libraries were prepared using the NEBNext® Ultra™ RNA Library Prep Kit for Illumina® (NEB, Ipswich, MA, USA) following the manufacturer’s recommendations, and index codes were added to tag the sequences. Finally, the libraries were sequenced on an Illumina HiSeq platform, and paired-end reads were generated.

### *De novo* transcriptome assembly and annotation

Clean reads were produced by removing reads containing adapters, reads containing poly-N sequences and low-quality reads from the raw reads. Simultaneously, the Q20, Q30, GC content and sequence duplication levels of the clean data were calculated using RNA-QC-Chain (Zhou et al., 2018). Transcriptome assembly was accomplished using Trinity (Grabherr et al., 2011) with default settings. To annotate the assembled unigenes, we performed BLASTX searches with *E*-values less than 1E−5 and 1E−3 against the NR (NCBI nonredundant protein sequences), NT (NCBI nonredundant nucleotide sequences), Pfam (protein family), KOG/COG (clusters of orthologous groups of proteins), Swiss-Prot (a manually annotated and reviewed protein sequence database), KO (Kyoto Encyclopedia of Genes and Genomes (KEGG) ortholog database), GO (gene ontology) databases.

### Differential gene expression analysis

Differential expression analysis of gill tissue was performed using the DESeq R package (1.10.1) (Anders & Huber, 2010), which provided statistical routines for determination of differential expression from gene expression data using a model based on the negative binomial distribution. The resulting *P*-values were adjusted using Benjamini and Hochberg’s approach for controlling the false discovery rate (FDR). Genes with adjusted *P*-values < 0.05, as identified by DESeq, were designated as being differentially expressed.

GO enrichment analysis of the DEGs was performed by using the GOseq R packages based on Wallenius’ noncentral hypergeometric distribution (Young et al., 2010), which can adjust for gene length bias in DEGs. We used KOBAS (Mao et al., 2005) software to test the statistical enrichment of DEGs in KEGG pathways.

### Quantitative real-time PCR (qRT-PCR) validation

To validate the RNA-Seq results, quantitative real-time PCR (qRT-PCR) was carried out for the four molting stages (stage A, stage C, stage D and stage E). The gills of three crabs in each molting stage were collected for RNA isolation by the TRIzol method as described above. cDNA was synthesized using the PrimeScript™ RT Reagent Kit (TaKaRa, Kusatsu, Japan) according to the manufacturer’s protocol. Nine significantly expressed genes were selected for qRT-PCR assays. PCR primers designed based on the assembled transcriptome sequences are listed in Table S1. The qRT-PCR analysis was performed using an IQ5 system (Bio-Rad, Hercules, CA, USA) with the SYBR® Premix Ex Taq™ Kit (TaKaRa, Kusatsu, Japan) according to the manufacturer’s instructions. The expression levels of each gene were normalized to those of β-actin (β-actin-f: 5′-AGTAGCCGCCCTGGTTGTAGAC-3′; β-actin-r: 5′-TTCTCCATGTCGTCCCAGT-3′). The primers (Supplemental Information 2) used for qRT-PCR were designed based on the specific gene sequences (Supplemental Information 3). All of the real-time PCR experiments were performed in three biological replicates, and the average threshold cycle (Ct) was calculated with the 2^−ΔΔ*Ct*^ method (Muller et al., 2002). The relative expression of the target genes between groups was statistically tested by ANOVA followed by the *T*-test (*P* < 0.05).

## Results

### *De novo* transcriptome assembly and annotation

From 12 gill samples at four molting stages, namely, postmolt, intermolt, premolt and ecdysis, we obtained a total of 1,712,109,230 clean reads from 1,826,272,782 raw paired-end reads (93.75%) after quality trimming using QC-Chain. These reads were de novo assembled to obtain 246,232 unigenes, which had a mean length of 851 bp and an N50 of 1,245 bp (Table 1). The length distributions of all the unigenes are shown in Fig. S1. The annotation result and accession number of all the unigenes (or referenced genes) are shown in Supplemental Information 1. To annotate the unigenes obtained, we performed BLASTX searches with *E*-values less than 1E−5 and 1E−3 against the NR, NT, Swiss-Prot, KOG, GO, KO and Pfam databases. We annotated 10,108, 29836, 52,963, 38,204, 68,664, 25,157 and 67,612 unigenes based on these databases, respectively. Finally, a total of 86,634 unigenes (35.18% of the total unigenes) were annotated against at least one database (Table 1).

**Table 1:** Statistics of *E. sinensis* transcriptome sequencing, assembly and annotation.

Sequencing	Raw reads	1826272782	
	Clean reads	1712109230	
	Number of Unigenes	246232	
Assembly	Total length (nt) of total unigenes	209633565	
	Mean length (nt) of total unigenes	851	
	N50 (nt) of total unigenes	1245	
		Number of Unigenes	Percentage (%)
Annotation	Annotated in NR	10,108	4.1
	Annotated in NT	29,836	12.11
	Annotated in KO	25,157	10.21
	Annotated in SwissProt	52,963	21.5
	Annotated in PFAM	67,612	27.45
	Annotated in GO	68,664	27.88
	Annotated in KOG	38,204	15.51
	Total Unigenes annotated	86,634	35.18

### Differentially expressed genes (DEGs) in the gills of *E. sinensis* during the molting process

**Figure 1:**
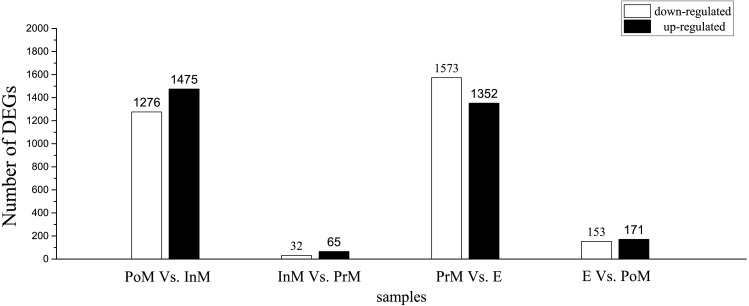
Differentially expressed genes (DEGs) between adjacent molting stages in *E. sinensis*. PoM, postmolt; InM, intermolt; PrM, premolt; E, ecdysis.

To gain insight into global gene expression levels in the gills during the molting process, we performed pairwise comparisons of gene expression between consecutive molting stages. Using log_2_ (fold change) >1 and FDR <0.005 as thresholds, a number of genes were found to be significantly upregulated in postmolt compared to intermolt (1,475), in intermolt compared to premolt (65), in premolt compared to ecdysis (1,352), and in ecdysis compared to postmolt (153). The corresponding numbers of downregulated genes were 1,276, 32, 1,573 and 171, respectively (Fig. 1).

To reveal the associations of gene expression with function and biological/metabolic pathways, we performed enrichment analysis of DEGs via both GO and KEGG annotation. Comparison of the four molting stages revealed that DEGs were enriched (corrected *P*-value < 0.05) in 68 (postmolt to intermolt) and 140 (premolt to ecdysis) GO terms (Table S1) and 21 (postmolt to intermolt), 48 (premolt to ecdysis) and 2 (ecdysis to postmolt) KEGG pathways (Table S2). Enrichment was not observed in either GO terms or KEGG pathways for DEGs between the intermolt and premolt stages.

**Figure 2:**
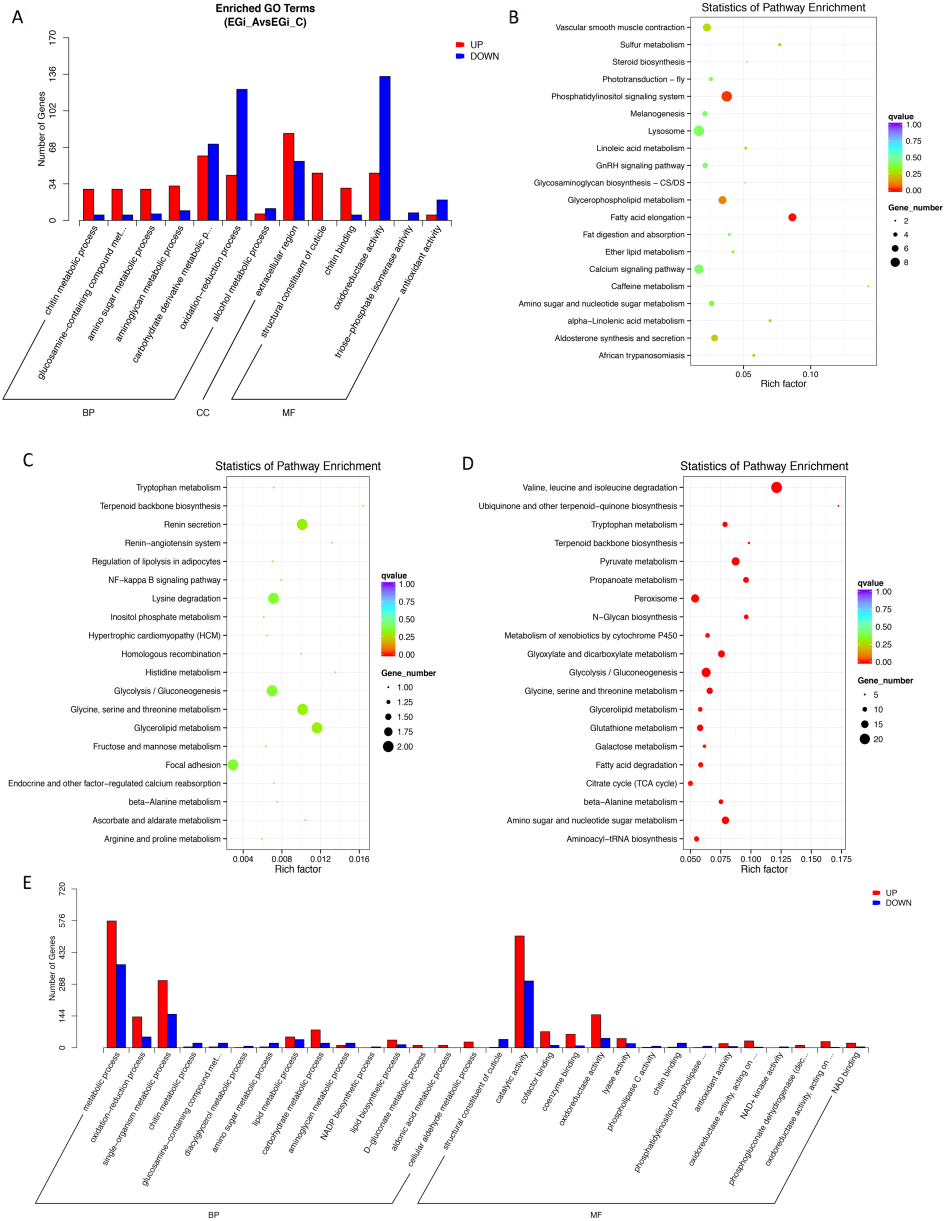
Enrichment of Differentially expressed genes (DEGs) between the key molting stages in GO and KEGG annotation (corrected *P*-value < 0.05). MF, Molecular Function; BP, biological process; CC, cellular component. (A) Enriched GO terms in A compared to C. (B) Enriched KEGG in A compared to C. (C) Enriched KEGG terms in C compared to D. (D) Enriched KEGG terms in D compared to E. (E) Enriched GO terms in D compared to E. A, postmolt, C, intermolt, D, premolt, E, ecdysis.

Specifically, during the postmolt stage, the upregulated genes were enriched in the GO biological process (BP) category of “chitin metabolic process” and the molecular function (MF) categories of “structural constituent of cuticle”, “chitin binding” and “alcohol O-acetyltransferase activity” (Fig. 2A, Table S1). All of these GO terms are associated with chitin metabolism, involving three chitin synthase genes, two chitinase genes, one chitin deacetylase gene and a number of genes that contain chitin-binding or other chitin metabolism-related domains. For KEGG pathways, the upregulated genes were found to be enriched in several lipid-related metabolic pathways, such as “fatty acid elongation”, “glycerophospholipid metabolism” and “sulfur metabolism”. Three signaling pathways were also enriched, including the “phosphatidylinositol signaling system”, the “calcium signaling pathway” and the “GnRH signaling pathway”. Other enriched pathways included “amino sugar metabolism”, “vascular smooth muscle contraction”, “caffeine metabolism”, “aldosterone synthesis and secretion” and “lysosome” (Fig. 2B, Table S2).

The highly expressed genes in the intermolt stage were mainly associated with the KEGG pathways “glycolysis/gluconeogenesis”, “metabolism of histidine”, “metabolism of amino acid”, “metabolism of glutathione”, “metabolism of glycerolipid”, etc. (Fig. 2C, Table S2). There were much fewer DEGs between intermolt and premolt than between other pairs, and the DEGs were enriched in neither GO nor KEGG annotations. Nevertheless, several genes exhibited significantly higher expression in the premolt stage than in the intermolt stage, such as those encoding rapamycin-insensitive companion of mTOR (*rictor*), dynactin subunit 1 (*dctn1*), spastic paraplegia 7 (*spg7*), the Ras-related protein Ral-A (*rala*) and cathepsin K (*ctsk*). Interestingly, the functions of these genes may be associated with the regulation of cytoskeleton formation.

The up-regulated genes in the ecdysis stage compared to the premolt stage were enriched in GO terms in the BP category of “chitin and amino sugar metabolic process”, which contained genes encoding chitinase 1, 2 and 3. In the MF category, “structural constituent of cuticle” contained 24 genes encoding cuticle proteins with high expression levels in this stage (Fig. 2E, Table S1). In the KEGG annotations, the highly expressed genes were enriched in “aldosterone synthesis and secretion”, “gastric acid secretion”, “salivary secretion”, “melanogenesis”, etc. A number of signaling pathways were also enriched, such as “phosphatidylinositol signaling system”, “GnRH signaling pathway”, “retrograde endocannabinoid signaling”, and “oxytocin signaling pathway” (Fig. 2D, Table S2). We also observed high expression levels of the genes encoding EcR and ecdysone-induced protein 75B, isoform B (Eip75b), which are thought to be important regulatory genes genes involved in the molting cycle of *E. sinensis*.

**Figure 3:**
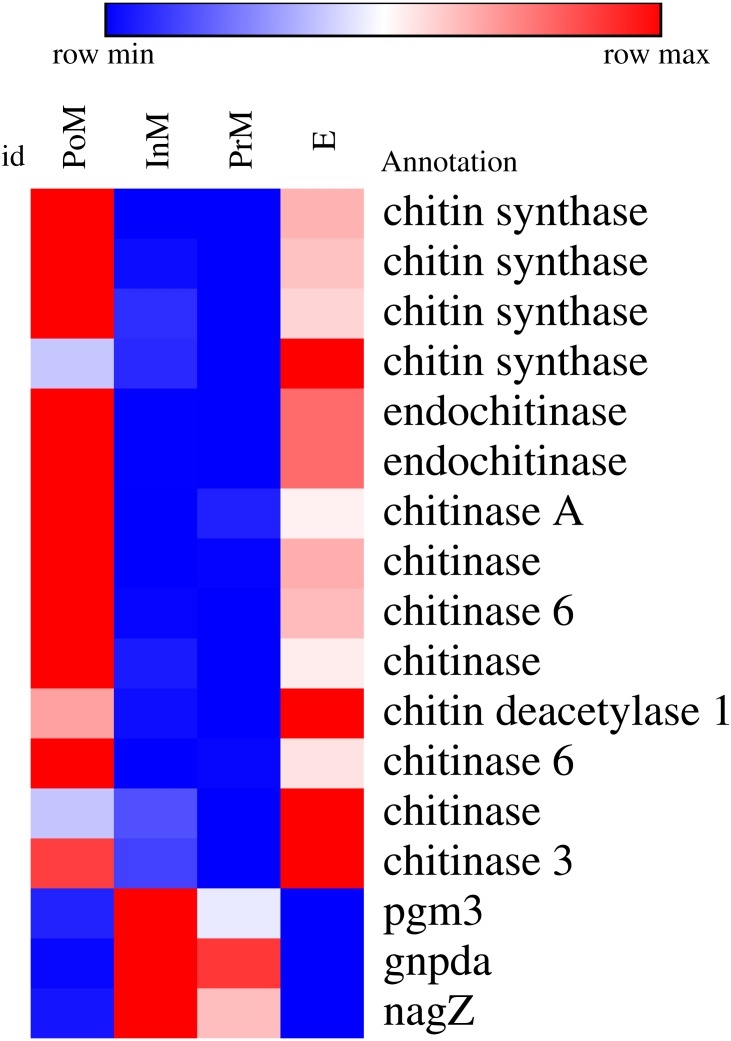
Heatmap of some differentially expressed genes (DEGs) involved in chintin metabolism in key molting stages in *E. sinensis*, including postmolt (PoM), intermolt (InM), premolt (PrM) and ecdysis (E). Colored keys represent the fold changes (log_2_ transformed counts) of gene expression between adjacent molt stages. Red represents up-regulation and blue represents down-regulation. Each column represents a molting stag and each row represents a DEG.

Chitin, a linear homopolymer of β 1–4-linked N-acetylglucosamine residues, is a major component of exoskeletal scaffolds of crustaceans. We found that the metabolism of chitin is delicately regulated at the transcript level during different stages of molting. We found that genes involved in chitin metabolism exhibited distinctly different expression patterns in different molting stages (Fig. 3). Genes encoding chitin synthase, endochitinase, chitinase A, chitinase 3, chitinase 6 and chitin deacetylase 1 were upregulated during the postmolt and ecdysis stages, and we detected high expression levels of chitin synthase genes, which correlated with the development and accumulation of the chitinous cuticular layer. However, the genes encoding phosphoglucomutase 3 (PGM3), glucosamine-6-phosphate deaminase (GNPDA) and glucosamine glycoside hydrolase (nagZ) exhibited greater upregulation during the intermolt and premolt stages than during other stages. All of these genes were associated with glucosamine glycoside and chitin synthesis.

Meanwhile, to explore the specific function of gill in the molting process, the different expression genes (DEGs) with the following pattern were focused on: these genes expressed at a higher level in the ecdysis, a lower level in the postmolt stage compared to the ecdysis stage, a very low expression in intermolt stage, a relatively higher in the premolt stage and reach the expression level peak in the next ecdysis stage. These genes up-regulate with the beginning of molting (in Premolt stage) and reach the top in the ecdysis stage, and barely expressed in the intermolt stage. In total, 56 genes expressed with the above pattern including tetraspanin-18, trypsin, ATP synthase (E/31 k Da) subunit, cadherin domain, transcription cofactor vestigial-like protein 4 and other 50 genes without referenced genes. Interestingly, the GO annotation of the most of 50 genes were “integral component of membrane” of cellular component. These genes were involved in the transmembrane signaling.

### Quantitative PCR validation

**Figure 4:**
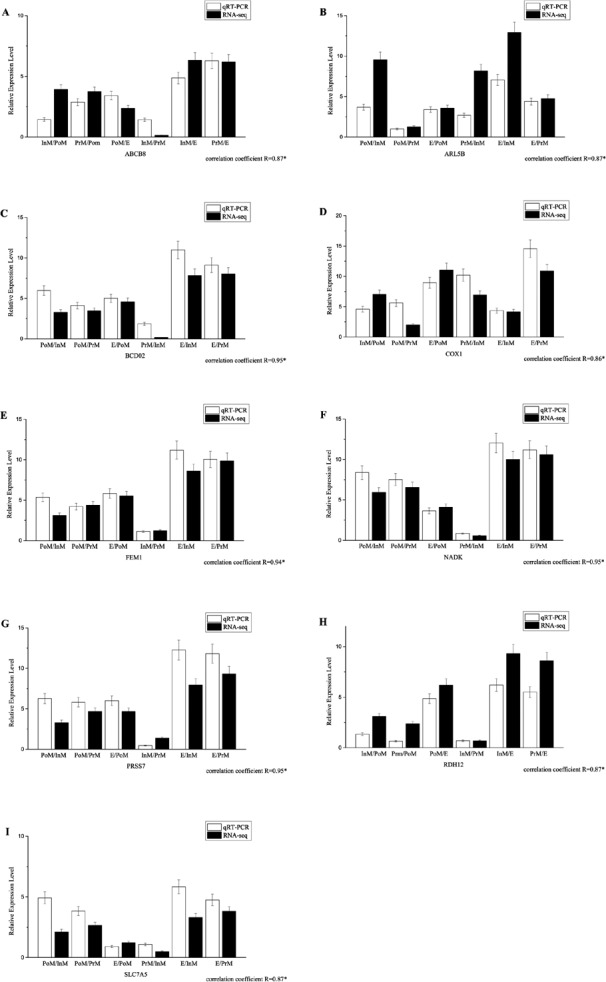
The expression pattern of genes in key molting stages in *E. sinensis*, including postmolt (PoM), intermolt (InM), premolt (PrM) and ecdysis (E) detected by the qRT-PCR and RNAseq data. Comparison of relative fold changes between RNA-seq and qRT-PCR results among the different group comparison. Fold changes are expressed as the ratio of gene expression in one molting stage to the other molting stages as normalized with β-actingene, while ‘qRT-PCR’ means the expression profile detected by qRT-PCR method and ‘RNA-seq’ means the transcriptome data.

All the primers, which were designed based on the assembled contig or unigenes from the transcriptomic data, were validated by PCR and traditional sequencing. Quantitative PCR experiments were conducted to validate the expression patterns of 9 selected genes, such as nadk, abcb8 and slc7a5 (Fig. 4, primer sequences are provided in Supplemental Information 2). The transcript levels determined by qRT-PCR were consistent with those determined by RNA-Seq. The results for the quantitative PCR experiments for all the selected genes demonstrated that the de novo transcriptome assembly and calculated expression levels were accurate. There was significant correlation between the two methods, with coefficients ranging from 0.86 to 0.97. Our results showed that the expression patterns of genes in the transcriptome could accurately reflect the gene expression profiles in individuals (Fig. 4).

## Discussion

Molting is essential for growth and development of crustaceans, which occurs several times during the life cycle of *E. sinensis*. Knowledge of the transcriptional regulation of genes associated with the molting process will provide an important and essential understanding of the physiological regulation of molting. The crustacean gill directly contacts the external water environment, playing important roles in respiration, osmotic regulation, ion-transportation and pathogen defense. In the molting cycle, the gill also undergoes shell extrication and formation as the exoskeleton. We analyzed the genome-scale gene expression profiles of *E. sinensis* gills during all the stages of molting, including postmolt, intermolt, premolt and ecdysis, using RNA-Seq and revealed the associations between the DEGs and the morphological and biochemical changes that occur during the different molting stages. Our finding may be helpful to provied a genetic foundation in the gill during molting cycle.

In the postmolt stage, feeding is paused, and the crab is in a relatively quiescent state. Still, many upregulated genes were involved in several energy metabolism-related pathways, which is similar to the transcriptomic results in hepatopancreas in this stage (Huang et al., 2015). For example, several highly expressed genes were associated with glycerophospholipid metabolism, such as secretory phospholipase A2 (pla2g), glycerol-3-phosphate dehydrogenase (*gpd1*) and diacylglycerol kinase (*dgk*). Seven genes encoding elongation of very long chain fatty acids protein 7 (ELOVL7), which is responsible for fatty acid elongation, were also overexpressed, suggesting that energy might be provided by utilization of stored lipids in this stage. Sulfur metabolism is also a form of energy metabolism, and the sulfide:quinone oxidoreductase (*sqor*) and sulfur dioxygenase (*sdo*) genes were overexpressed. SQOR catalyzes the first step in hydrogen sulfide metabolism and produces the metabolite sulfane sulfur. The function of SDO is to oxidize persulfide to sulfite using O^2^ and H_2_O. Both SQOR and SDO are oxidoreductases that participate in the hydrogen sulfide metabolism and play essential physiological roles in both sulfide detoxification and energy transduction.

Intermolt is the stage in which dissolution of the old cuticle occurs, accompanied by synthesis of the new shell. During this stage, the crab consumes large amounts of food for accumulation of nutrition, energy and mineral elements in preparation for molting and formation of the new cell. We found that the upregulated genes were strongly enriched in a variety of metabolic processes that are essential for growth and development. For instance, thirteen genes involved in glycolysis were upregulated, such as phosphoglucomutase (*pgm*), glucose-6-phosphate isomerase (*pgi*), fructose-bisphosphate aldolase (*fba*), triosephosphate isomerase (*tpi*), phosphoglycerate kinase (*pgk*), 2,3-bisphosphoglycerate-dependent phosphoglycerate mutase (*pgam*), enolase (*eno*) and aldehyde dehydrogenase (*aldh*), indicating improvement of conversion of glucose to pyruvic acid, which acts as an energy supplier in cells. Several highly expressed genes, such as *pgi, pgam* and *pgk* showed similar regulation in hepatopancreas (Huang et al., 2015), indicating a synchronic regulation in the glycolysis pathway in gill and hepatopancreas. In glycerolipid metabolism, genes encoding triacylglycerol lipase (*lip*) and diacylglycerol kinase (*dgka*) were upregulated. LIP catalyzes the hydrolysis of triacylglycerol, producing diacylglycerol. DGKA acts as a regulator that competes with protein kinase C for the second messenger diacylglycerol and plays an important role in regulation of the regeneration of phosphatidylinositols and phosphatidate. Activation of glycerolipid metabolism is correlated with the accumulation of lipids, which provide energy and are thus crucial for successful ecdysis.

Premolt is the stage in which the crab undergoes apolysis or separation of the old exoskeleton from the underlying epidermal cells. We found that several of the genes that were overexpressed at this stage were associated with the cytoskeleton. *Rictor* encodes a subunit of mTORC2, which is implicated in the control and maintenance of the actin cytoskeleton. The protein encoded by *dctn1* is the largest subunit of dynactin, which regulates microtubule stability by promoting microtubule formation, nucleation and polymerization (Ayloo et al., 2014). *Rala* encodes a protein belongs to the Ras family of proteins in the small GTPase superfamily. This protein mediates transmembrane signaling and was reported to contribute to the regulation of microtubule and actin cytoskeletal reorganization (Papini et al., 2015). *Ctsk* encodes a lysosomal cysteine proteinase that is involved in bone remodeling and resorption and is highly expressed in activated osteoclasts (Costa et al., 2011). We propose that the high expression levels of these genes before ecdysis may indicate upregulation of cell proliferation and may aid the formation of a new layer and exoskeleton in preparation for digestion of the old integumentary shell. We also found that in the premolt stage, the regulated genes in gill were different from those identified in hepatopancreas, which were enriched in ecdysone hormone regulation (Huang et al., 2015).

Ecdysis is a short stage in which the crab extricates itself from a crack in the old exoskeleton via movement. In addition, in this stage, the crab uptakes water, leading to rapid expansion of the body. However, the mechanism underlying the physiological reactions associated with this rapid process are largely unknown. We found that upregulated genes at this stage were enriched in some related pathways, including the metabolic pathways of aldosterone, saliva and gastric acid. All of the metabolic pathways are associated with homeostasis of plasma sodium (Na^+^), potassium (K^+^) and chloride (Cl^−^) ions levels. Genes encoding PLC β, CALM and PKC were shared among these pathways and were found to be upregulated. These findings indicated that the regulation of salt levels and hemolymph pressure within the body might be enhanced in the premolt stage, helping the crab adapt to variations in body fluid status immediately after shelling. Variations in the blood pressure, combined with bodily movements, might also contribute to the appearance of the crack that allows the organism to extricate itself.

Another pathway that was enriched in the ecdysis stage was melanogenesis (Pillaiyar, Manickam & Jung, 2017), which is a complicated cellular process that produces melanin, leading to pigmentation. Melanin also plays a substantial role in protection of the skin against the harmful effects of ultraviolet radiation and oxidative stress from various environmental pollutants. The high transcript levels of genes involved in melanogenesis might facilitate increased melanin production (Hearing & Tsukamoto, 1991; Videira, Moura & Magina, 2013), accounting for the changes in pigmentation and providing protection to the crab during ecdysis and in the following stage. Overexpressed genes involved in melanogenesis included *plcb, pkc, calm*, mitogen-activated protein kinase 1 (*map2k1*) and adenylate cyclase 5 (*adcy5*).

In addition, we found that the metabolism of some specific compounds, such as chitin, was finely regulated at the transcript level during different molting stages. Chitin, a linear homopolymer of β 1–4-linked N-acetylglucosamine residues, is a major component of the exoskeletal scaffolds of crustaceans. We found that genes involved in chitin metabolism exhibited distinct expression patterns in different molting stages (Fig. 4). During the postmolt stage, we detected high expression levels of chitin synthase genes, which were correlated with the development and accumulation of the chitinous cuticular layer. A similar expression pattern for chitin synthase was also observed in the white leg shrimp *L. vannamei* (Gao et al., 2017; Rocha et al., 2012). On the other hand, we also observed transcriptional upregulation of a few genes encoding chitinase and chitin deacetylase, which catalyze chitin degradation. Previous studies on the fiddler crab *Uca pugilator* (Muller et al., 2002) have shown a similar expression pattern for chitinase (Rocha et al., 2012). A possible explanation for the observed expression patterns is that chitin degradation and modification may be involved in the remodeling of chitinous scaffolds during the synthesis of these scaffolds, and the breakdown products of chitin could be reutilized for the formation of the new cuticle. Moreover, before the newly formed exoskeleton hardens, the crab is vulnerable to infection. Chitin synthase and chitinase have been reported to have immune functions and can participate in defense mechanisms to protect the crab from infection. In the intermolt stage, we identified three highly expressed genes involved in amino sugar and nucleotide sugar metabolism, including genes encoding beta-N-acetylhexosaminidase (*nagZ*) (Wang et al., 2006), glucosamine-6-phosphate deaminase (*gnpda*) (Comb & Roseman, 1956; Comb & Roseman, 1958) and phosphoacylase glucosamine mutase (*pgm3*) (Cheng & Carlson, 1979). These enzymes catalyze a series of intermediate steps in the metabolism of the degradation product and precursor of chitin. During molting, we observed high expression of several genes encoding chitinases (chitinase A, chitinase 3 and chitinase 6) in gill. Chitinase and chitinase-like proteins from crustaceans can be classified into at least four groups (Huang et al., 2010) or six groups (Salma et al., 2012) based on phylogenetic analysis and domain organization. In *L. vanname*, only Chitinase (2, 5 and 6) could be detected in general gill. Meanwhile, in *Pandalopsis japonica,* none of the known Chitinase could be detected in the general gill tissue. In oriental river prawn *Macrobrachium nipponense*, six genes coding chitinase (1A, 1B, 3A, 3B, 3C and 4) were identified. However, only chitinase 4 was detected to express in gill and may just have a supporting function during the molting process (Zhang et al., 2014). The other Chitinase 1A, 1B and 3B have pivotal roles in the molting cycle but failed to be detected in the gill. In present study, chitinase 3 and chitinase 6 which showed a high expression level in PoM and E molting process and a very low level in InM and PrM process were detected in the gill. This result may suggest that the potential role of chitinase 3 and chitinase 6 in the molting cycle. Interestingly, in the study of *L. vannamei* (Guo, Xian & Wang, 2016) under the stress of nitrite, the Chitinase showed a down-regulated expression pattern. More intensive studies are needed to clarify the structure, classification and function of the chitinase genes in gill of *E. sinensis.* The highly expressed genes, namely, phosphoglucomutase 3 (PGM3) (Mariappa et al., 2011), glucosamine 6-phosphate deaminase (GNPDA) (Comb & Roseman, 1958) and glucosamine glycoside hydrolase (nagZ) (Kitaoka, 2015), were associated with glucosamine glycoside, and these important genes were associated with chitin synthesis.

These genes which were up or down regulated with the whole molting cycle may reflect the specific function of gill in the molting process. Unfortunately, most of these genes cannot be identified for lacking references genes but can be identified by GO analysis. Many genes are involved in the transmembrane signaling. The different expression gene tetraspanin-18 was a member of tetraspanin super family (Hemler, 2005). Tetraspanin super family is cell–surface proteins, and often hidden by a canopy of tall glycoprotein neighbours in the cell membrane. Tetraspanin protein functioned in cell biology, signaling and biochemistry (Hemler, 2001; Maecker, Todd & Levy, 1997). Tetraspanins protein played role as regulators of protein trafficking (Berditchevski & Odintsova, 2007). Tetraspanin-18 gene up-regulated with the beginning of molting (in Premolt stage) and reached the top at the ecdysis stage, and barely expressed at the intermolt stage. In other crustacea, the tetraspanin genes showed a regulated expression pattern in the hepatopancrea of river prawn *Macrobrachiu mnipponense* (Yu et al., 2019) and *L. vannamei* (Guo, Xian & Wang, 2016) under the stress of nitrite. The molting cycle is regulated by many environmental factors, such as temperature and salinity. The gill is a tissue that directly contact with the water, sensing changes of the environmental factors and performing osmoregulatory functions (Zhou & Jiang, 2004). The expression variations in the tetraspanin-18 gene indicated that it may play an important role in the beginning of molting cycle, which might be regulated by the stress of salinity. In addition, the tetraspanins and tetraspanins-like proteins are important effector genes of lysosome integration membrane glycoprotein (LIMP) in the lysosomal pathway, which is involved in cell apoptosis. Tetraspanins proteins functioned in multiple physiological and pathological processes such as the cell development, cell adhesion, cell motility or the probably cell differentiation, which play critical roles (Hemler, 2005; Levy & Shoham, 2005; Trikić et al., 2011; Yeh & Klesius, 2010). Meanwhile, the other genes such as the trypsin, ATP synthase (E/31 k Da) subunit, cadherin domain and transcription cofactor vestigial-like protein 4 may function as the coworker or provide assistance in the process of molting. The role of these genes is still unknown, more studies, such as the discovery of signal which tiger the up-regulation of these genes or the role of Tetraspanin-18 gene in the whole molting cycle, should be performed.

## Conclusions

Here, we report the transcriptomic variations in the gills of *E. sinensis* in different molting stages. The identification of DEGs with functional implications provides insights into the underlying regulatory mechanisms of the molting process. This information also provides genomic resources for improvement of the growth and development performance of *E. sinensis*.

##  Supplemental Information

10.7717/peerj.7182/supp-1 Figure S1 Statistics of the size distributions of unigenes

10.7717/peerj.7182/supp-2 Supplemental Information 1 Annotation of *E. sinensis* unigenes

10.7717/peerj.7182/supp-3 Supplemental Information 2 List of the primers for qRT-PCR

10.7717/peerj.7182/supp-4 Supplemental Information 3 List of the gene sequences for qRT-PCR

10.7717/peerj.7182/supp-5 Table S1 GO annotation of *E. sinensis* DEGs

10.7717/peerj.7182/supp-6 Table S2 KEGG annotation of *E. sinensis* DEGs

## Additional Information and Declarations

### Data Availability

The following information was supplied regarding data availability: The raw sequencing reads are available at NCBI: BioProject ID PRJNA472198.
