# Supplementary figures and images for: Transcriptomic analysis of gills provides insights into the molecular basis of molting in Chinese mitten crab (Eriocheir sinensis)
Source: PeerJ. 2019 Jun 28;7:e7182. doi: 10.7717/peerj.7182 (PMC6601604; doi:10.7717/peerj.7182)

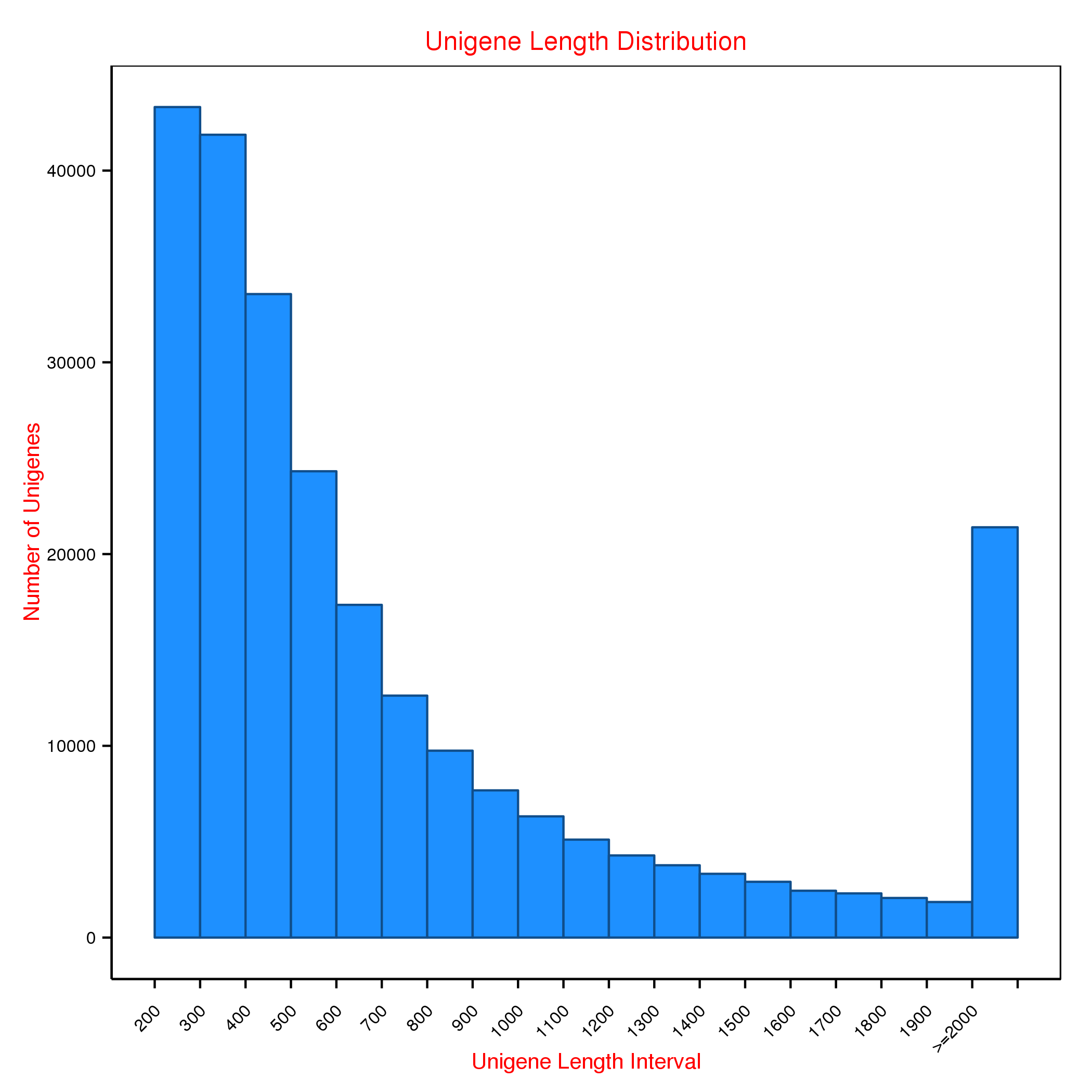

Supplement: Figure S1 [file peerj-07-7182-s001.png]
